# Experimental Study of the Process of Repair

**Published:** 1876-12

**Authors:** I. N. Danforth

**Affiliations:** Lecturer on Pathology in Rush Medical College


### EXPERIMENTAL STUDY OF THE PROCESS OF REPAIR.

By I. N. DANFORTH, M. D.,

Lecturer on Pathology in Rush Medical College.

(Read before the Chicago Medical Society, August 7th, 1876.)

The successful study of the healing process, in all its stages, has always been a matter of great difficulty. Indeed, I am not aware that any observer has heretofore attempted making consecutive microscopic obversations at brief intervals, of the *same wound in living tissue*, from the time of its infliction to its complete repair; or of presenting figures drawn from nature, illustrative of this process. Let any one glance at the figures in "Billroth's Surgical Pathology" which are designed to illustrate the process of repair; it is evident that they are, in the main, diagramatic, and that they illustrate what is *supposed* to occur, rather than what is *known* to occur. And, in general, the difficulties standing in the way of such studies are simply insurmountable. The time required for the observation of the various steps of such a process necessarily occupies several days, and demands very careful, delicate and nearly constant attention from the operator.

It is requisite in the first place to find a transparent and vascular[1] tissue which can be retained in one constant position, and *yet be kept alive*, for several consecutive days. Again,

[1] The following account does not, of course, apply to non-vascular parts.

the animal which exercises ownership over this living tissue, must somehow be induced to remain perfectly passive and non-resistant during the entire period of time needful for the experiment; one single violent attempt to escape after the experiment is fairly under way, would be quite sufficient to spoil the whole. Lastly, some anæsthetic or paralyzing agent must be employed which will produce quietude without causing death. It will be seen at once that this series of conditions involves some difficulties which must be surmounted.

Some time ago, Henry W. Fuller, Esq., of this city, President of the Illinois State Microscopical Society, requested me to contribute something in the way of a popular, illustrated lecture, for the entertainment (if not profit) of the Society over which he presides. Having studied inflammation many times experimentally upon the web, tongue and mesentery of the curarized frog, and having thus familiarized myself somewhat with the use of this singular poison, it occurred to me that it might be possible to inflict a small wound in the frog's tongue, and then watch the progress of the process of repair. But in order to render the fruits of such a study practically useful and available, it was also necessary that the changes which took place from day to day be fixed in some permanent form so that *others* could see them. This would render the services of an artist indispensable. When I communicated my plan to Mr. President Fuller, he at once authorized me to proceed, and draw on him for the required expense. As a result of this characteristic liberality on the part of Mr. Fuller, I am now in possession of ten "Artographic Lantern Slides," which very beautifully and faithfully represent as many stages of this most interesting, and, as I believe, novel experiment. And here it is proper that I should briefly explain the "Artographic" process of illustration, as well as acknowledge my obligations to its originator, Dr. R. U. Piper, of this city.

The "Artographic Lantern Slide," is essentially a Photographic Lantern Slide, colored so as to quite perfectly imitate nature in many instances and enable us in all cases to differentiate the various tissues and structures by conventional colorings. In the present instance Dr. Piper made careful drawings by aid of the camera lucida, as often as any change worth recording took place in the wound. These drawings, which were of course mathematically correct and all drawn to the same scale, were then transferred to glass by a process of etching, the application of which is original with Dr. Piper; the etchings were then photographed by Mr. P. B. Green, of this city, and the resulting transparencies were colored by Dr. Piper's own hand.

It seems to me that this beautiful process must come extensively into use for the purpose of anatomical and histological demonstration, because it appears to supply a real want. The ordinary photographic transparencies of anatomical structures are wanting in sharpness and definition, and it is impossible to indicate different tissues by the aid of different colors. These important *desiderata* are quite perfectly supplied by Dr. Piper's process.

If the reader is not already discouraged by this somewhat prolix explanatory introduction, I will now ask him to follow the course of the experiment from the date of the infliction of the wound, to its complete repair. On the evening of February 23, 1876, a healthy, active, medium sized frog was brought under the influence of curare, by the subcutaneous injection of about $\frac{1}{2000}$th of a grain.[1] The animal was then placed upon a frog plate, the tongue gently drawn out and its edges pinned to a piece of perforated cork, which was fitted to the opening in the frog plate; the perforation in the cork being covered by a slip of glass, upon which, of course, the tongue rested. Especial care was taken not to put the tissue of the tongue so much upon the stretch as to materially interfere with the free circulation of the blood, or the action of the blood-vessels.[2]

[1] I take pleasure in acknowledging my obligations to Lieut. Col. J. J. Woodward, M.D., of the Army Medical Museum, Washington, D. C. for a supply of genuine curare, without which this experiment would have been impossible.

[2] It must be remembered that the tongue of the frog is attached to the lower jaw in front, but is free posteriorly. In capturing insects the organ is thrown out of the mouth and drawn in again with inconceivable rapidity, the game being involved in a glutinous secretion.

A minute wound was now made through the tongue by means of a pair of sharp pointed scissors, lengthwise of the organ, or in the direction of the muscular fibres; care being taken to select an islet of tissue which was least vascular, and in which the few vessels were quite small. The frog was now placed upon the stage of the microscope, the changes of the next half hour closely watched, and a faithful camera drawing made by Dr. Piper; the first drawing being executed at 9 o'clock, on the evening of February 23d, or about one hour after the wound was made. The appearances at this time were as follows: the wound was rather more than one-sixteenth of an inch in length; its form was nearly oval, the edges having been drawn apart by the contraction of the muscular fibers. The bottom or floor of the wound was covered by a pretty firm blood clot; the blood having slowly oozed from the cut ends of the minute bloodvessels which occupied the track of the wound. In watching the escape of this blood from the vessels, I noticed this singular phenomenon, namely: that the escaped blood globules, or more properly the current of extra vascular blood, moved in compact and orderly columns across the floor of the wound; that is, after the vessels were cut and hæmorrhage had ensued, the escaped blood did not flow in *all* directions in a helter skelter disorderly manner, but formed itself into different columns, representing the different vessels from which it flowed, and these several columns advanced in regular order, with but little variation in their course, until two streams chanced to encounter each other, when the larger and stronger stream would generally bear away the smaller and weaker one.

In another hour all oozing of blood had ceased, coagula had formed in the wounded vessels up to the next collateral branch, and the condition of the wound and its immediate surroundings was that of apparent "masterly inactivity." The tongue was now (10 o'clock) covered with a light compress of muslin wet in water, the frog's skin moistened, and the animal laid aside for the night.

February 24th, 9 A. M., (12 hours after injury,) circulation is now active in all parts of the tongue, except immediately arround the cut. In the immediate vicinity of the wound, the vessels are distended and gorged with blood, and stasis has occurred in the greater part of them. In some, however, the circulation is still wonderfully active and vigorous. The walls of the wound are mostly smooth and sharply defined, but from the (apparently) upper and lower border, a little bud, irregularly knotted, pale and terminating in a club-shaped extremity has commenced shooting out; the two buds (which are two masses of round cells) are almost opposite, and seem to be growing toward each other. "I have an impression," says the record which I made at the time, "that they are incipient blood vesels, and that I am about to witness the growth of new vessels." The floor of the wound presents, 1st, a few minute coagula, 2d, blood globules, both white and red, and 3d, a few pale, very minute fibers of fibrine. The changes were so slight, that it was not deemed necessary to make a drawing this morning.

February 25th, 9 A. M., (36 hours after injury.) "The little growing buds from which I expected so much, have undergone no further development since yesterday; in fact they have grown smaller." The minute fibers, on the floor of the wound have increased, and are interwoven in all directions, and in their interstices are many blood globules of both varieties. The surrounding vessels are enlarged. and somewhat tortuous; in vessels which show any movement of blood at all, the circulation is very active. Small coagula are seen at intervals along the border of the wound. The tissues surrounding the injury are becoming slightly cloudy, unlike the sharpness and clearness of the more remote strictures. On examining the tissues involved with No. 5 Hartnack, I can easily see that the cloudiness is due mainly to the migration of leucocytes, which are rapidly insinuating themselves into the[1] areolar and muscular interstices.

[1] I ought to have said before that the studies were chiefly made with Tolle's inch, and the "a" eye piece, a combination giving on my microscope about 85 diameters; the drawings were all made from this combination. The quarter inch was employed daily however, for the purpose of studying the minute changes.

February 26th, 9 A. M., (60 hours after injury.) The wound is considerably smaller; the cloudy appearance of the surrounding tissues has increased; the border of the wound is now quite thickly fringed with little buds or protrusions, which, upon being examined with a quarter inch, are seen to be composed almost exclusively of cells. In some of these growing buds, a minute blood-vessel, with its tiny blood stream, can be made out. These vessels are always in the form of loops, a single minute current flowing into the cell bud through one branch of the loop, and immediately returning through the other. Here, then, we have a granulation growing up under our eyes, under conditions which admit of its being quite freely observed in a leisurely and satisfactory manner.

The circulation in the vicinity of the wound is very active; the blood-vessels are dilated and tortuous; stasis is no longer present except in a few quite small vessels. The veins in the periphery of the microscopic field are enormously distended, and the blood stream is both very dark and very slow. In some of the vessels in sight, the jerky, interrupted movement of blood, so often described by authors as occurring in inflammation, can be very distinctly seen. Many blood globules are seen on the floor of the wound, but the net work of fibers spoken of in yesterday's record has nearly disappeared.

February 27th, 9 A. M., (84 hours after injury.) The area of the wound is no smaller than it was yesterday; in fact it seems a little larger. New vessels are multiplying around the wound, and new buds are pushing their way into the open space. The floor of the wound is now occupied by a thickly woven reticulated net work of minute fibers of coagulated fibrine, which have formed since the last observation. The interstices are filled by blood globules, both red and white. The circulation is very active, and the frog seems in good health.

February 28th, 9 A. M., (108 hours after injury.) The size of the wound has diminished very greatly since yesterday. No other marked change has taken place; the circulation seems neither more or less active than it was yesterday; new vessels are still forming; new granulations are springing up, and the size of the wound is rapidly diminishing.

February 29, 9 A. M., (132 hours after injury.) Several new blood vessels appear this morning, and the pre-existing ones, especially the veins are enormously dilated. The wound diminishes with great rapidity. The walls or "banks" of the wound now present a very curious and interesting appearance. At short intervals, little simicircular rills of blood are seen running along the shelving and somewhat precipitous "banks." Each little semicircle, or loop, is covered in by a dense layer of small round cells ; the deeper layer of cells assume a spindle form; thus new fibers are formed, and the wound is repaired.

March 1st, (156 hours after.) The cut is now reduced to a minute oval slit, and the floor is quite covered with minute newly formed fibers, interwoven in all directions. Meantime, new granulations are continually forming along the side-walls or banks of the injured part, and thus the solution of tissue-continuity is being repaired.

March 2d, (180 hours after.) With this record, the daily observation ceases. The wound is now practically closed, and its original area is nicely mapped out by the presence of new (cicatricial) tissue.

March 8th, (fourteen days after the infliction of the wound.) The frog was again curarized, the cicatrix examined, and a careful drawing made. It was easy to trace the exact extent of the injury by the lighter color of the new tissue. The cicatrix was entirely made up of new connective tissue; no regeneration of muscular tissue could be discovered. The new connective tissue appeared to be entirely the product of spindle-shaped cells, interwoven in all directions.

On a future occasion, the series of photographic pictures will be shown to the Society, projected on the screen, if an opportunity is afforded.

In conclusion, I desire briefly to call the attention of the Society to the lessons which the experiment has afforded:

*First*—And as it seems to me most important, is the demontion of the fact, that continuous experiments in physiology and pathology may be successfully and profitably carried on outside of special laboratories. When the frog is placed under the influence of curare, perfect quietude is insured, and the charge of cruelty no longer obtains. According to Dr. J. Burdon Sanderson, chloral may be made to answer the same purpose, from one to three grains in solution, being injected under the skin. The effect of chloral, however, is less permanent than that of curare; a dose of the former quiets the frog for six or eight hours only, while it takes fully twenty to twenty-four hours to recover from the influence of a single dose of curare.

*Secondly*—I was surprised at the frequent changes in the condition of the blood-clot in the wounded part, and at the frequency of the escape of fresh masses of blood. At first free hæmorrhage occurred, and the cavity of the wound was speedily filled with a coagulum. In a short time, the red globules had nearly disappeared, an intricate network of fine fibrinous fibres had formed, and in the interstices of this network, multitudes of mobile cells (*i. e.*, leucocytes) had accumulated. A few hours later, this clot had nearly disappeared, the fibers were wanting, except at the periphery of the wound, but a great number of leucocytes still remained and continued their wanderings over the floor of the wound. Several times while the tongue was under examination, the tenuous walls of the newly formed vessels would yield to the pressure of the blood stream. Hæmorrhage would occur, a fresh clot would form, only to disappear after an interval of a few hours. Meantime new fibers were forming between the periphery of the clot (or successive clots), and the inner border of the wound, so that the area of the wound was constantly growing smaller.

*Thirdly*—The constant and progressive dilatation of the larger vessels—especially the veins—in the immediate vicinity of the wound, was much greater than I anticipated. Some of the veins appeared like enormous channels or sinuses, excavated in the tissues, rather than actual vessels, with their proper tunics; and this condition of things remained for many days after the termination of the experiment.

The question as to the origin of the newly-formed fibers of the cicatrix came up, but I confess my inability to give a satisfactory answer.

Three sources are possible: 1st, they may be the product of the differentiation of the extravasated fibrine, or rather fibrine producing materials; 2d, they may be derived from the leucocytes, or from the progeny of the leucocytes; or 3d, they may be the result of the proliferation of the connective tissue corpuscles, situated in or upon the borders of the wound.

The discussion of this question, which is one of great pathological interest, would form a respectable paper of itself. As to the real facts, I am not yet prepared to express any decided opinions. If future experiments shall prove any more satisfactory, the result will be at the service of the Society.

